# Proof of Concept of Microbiome-Metabolome Analysis and Delayed Gluten Exposure on Celiac Disease Autoimmunity in Genetically At-Risk Infants

**DOI:** 10.1371/journal.pone.0033387

**Published:** 2012-03-14

**Authors:** Maria Sellitto, Guoyun Bai, Gloria Serena, W. Florian Fricke, Craig Sturgeon, Pawel Gajer, James R. White, Sara S. K. Koenig, Joyce Sakamoto, Dustin Boothe, Rachel Gicquelais, Deborah Kryszak, Elaine Puppa, Carlo Catassi, Jacques Ravel, Alessio Fasano

**Affiliations:** 1 Mucosal Biology Research Center, Center for Celiac Research and Departments of Pediatrics, Medicine and Physiology, University of Maryland School of Medicine, Baltimore, Maryland, United States of America; 2 Institute for Genome Sciences and Department of Microbiology and Immunology, University of Maryland School of Medicine, Baltimore, Maryland, United States of America; 3 Department of Pediatrics, Università Politecnica delle Marche, Ancona, Italy; Baylor College of Medicine, United States of America

## Abstract

Celiac disease (CD) is a unique autoimmune disorder in which the genetic factors (DQ2/DQ8) and the environmental trigger (gluten) are known and necessary but not sufficient for its development. Other environmental components contributing to CD are poorly understood. Studies suggest that aspects of gluten intake might influence the risk of CD occurrence and timing of its onset, i.e., the amount and quality of ingested gluten, together with the pattern of infant feeding and the age at which gluten is introduced in the diet. In this study, we hypothesize that the intestinal microbiota as a whole rather than specific infections dictates the switch from tolerance to immune response in genetically susceptible individuals. Using a sample of infants genetically at risk of CD, we characterized the longitudinal changes in the microbial communities that colonize infants from birth to 24 months and the impact of two patterns of gluten introduction (early vs. late) on the gut microbiota and metabolome, and the switch from gluten tolerance to immune response, including onset of CD autoimmunity. We show that infants genetically susceptible to CD who are exposed to gluten early mount an immune response against gluten and develop CD autoimmunity more frequently than at-risk infants in which gluten exposure is delayed until 12 months of age. The data, while derived from a relatively small number of subjects, suggest differences between the developing microbiota of infants with genetic predisposition for CD and the microbiota from infants with a non-selected genetic background, with an overall lack of bacteria of the phylum Bacteriodetes along with a high abundance of Firmicutes and microbiota that do not resemble that of adults even at 2 years of age. Furthermore, metabolomics analysis reveals potential biomarkers for the prediction of CD. This study constitutes a definite proof-of-principle that these combined genomic and metabolomic approaches will be key to deciphering the role of the gut microbiota on CD onset.

## Introduction

Celiac disease (CD) is a unique autoimmune disorder in that the key genetic components (HLA class II genes DQ2 and/or DQ8) are present in almost the totality of patients, the auto-antigen (tissue transglutaminase) has been identified, and, most importantly, the environmental trigger (gluten) is known [1]. Incomplete gluten digestion by intraluminal enzymes, changes in intestinal permeability and activation of innate immunity mechanisms are integral parts of the CD pathogenesis and all seem to precede the activation of the CD T-cell–mediated adaptive immune response leading to the autoimmune insult [2]. CD is strongly associated with specific HLA class II genes known as HLA-DQ2 and HLA-DQ8 located on chromosome 6p21. A multitude of non-HLA genes contribute to the CD genetic background, but each of them adds only a modest contribution to the disease development [3].

Gluten, the major protein component of wheat, with similar toxic proteins in rye and barley, represents the main environmental factor. The high proline content renders gluten proteins resistant to complete proteolytic digestion, leading to the accumulation of relatively large peptide fragments with a high proline and glutamine content in the small intestine triggering abnormal immune response in susceptible individuals [4]. The response is cooperatively mediated by both the innate and the adaptive immune systems. The adaptive response is due to gliadin-reactive CD4+ T-cells with consequent production of proinflammatory cytokines and specific anti-tissue transglutaminase (tTG) antibodies [5], [6]. The innate immune response in the intestinal epithelium is characterized by increased expression of interleukin-15 by enterocytes, resulting in the activation of intra-epithelial lymphocytes [7]. Furthermore, in CD, gluten causes CXCR3-mediated release of zonulin [8], a protein that enhances intestinal permeability by targeting the proteinase activator receptor 2 (PAR) and subsequent transactivation of epidermal growth factor receptor (EGFR) [9]. This loss of intestinal barrier function causes the uncontrolled passage of gluten peptides and other environmental antigens from the gut lumen to the lamina propria with their subsequent exposure to the gastrointestinal immune system. Detection of anti-gliadin antibodies (AGA) has been abandoned as a test for CD diagnosis due to their poor specificity for the disease. However, AGA of the class IgG has been previously reported as a possible biomarker of increased intestinal permeability [10], [11].

Long regarded as a gastrointestinal disorder of childhood, the disease is now more often diagnosed in adults than in children [12]. The reasons for this delayed onset of the disease remain undefined. It has been speculated that loss of gluten tolerance leading to immunological and mucosal changes typical of CD usually develops early in life, soon after the exposure to the environmental trigger (i.e., at weaning), while the onset of clinical manifestations of the disease can appear much later [1]. However, we have recently demonstrated that loss of gluten tolerance may occur at any time in life for reasons that are currently unclear [13]. Several data suggest that many aspects of gluten intake might influence the risk of CD occurrence and the timing of its onset, i.e., the amount and the quality of ingested gluten, together with the pattern of infant feeding and the age at which gluten is introduced in the diet. It is well established that infant nutrition in the first year of life is critical, as derangements of tolerance to food antigens can lead to food allergic disorders [14].

Still the environmental components other than gluten that favor CD development are thought to be numerous and poorly understood. Fewer than 10% of individuals with an increased genetic susceptibility develop clinical disease and most of them develop the condition many years after their first exposure to gluten. This suggests that, beside gluten, other environmental trigger(s) could be involved in the pre-autoimmune process. Several mechanisms that could lead to development of a gluten specific T-cell response have been proposed. For example, it is has been hypothesized that molecular mimicry between the EIB protein of adenovirus type 12 [15] or hyphal wall protein 1 of *Candida albicans* [16] and gluten could act as major factors that trigger the disease, but this remains speculative. It has been also proposed that a high frequency of rotavirus infections increases the risk of CD in childhood in genetically predisposed individuals [17]. The increased production of IFN-γ in anti-enteroviral immunity would lead to a shift toward Th1 responses leading to loss of tolerance for gluten [18]. Similarly, it has been postulated that the GI tract microbiota may play a major role in the pathogenesis of CD. For example, rod-shaped bacteria were frequently associated with the mucosa in CD patients, with both active and inactive disease, but not in controls [19], however a direct role for these bacteria or any others in disease development has not been established. Although intestinal infections might explain the break of tolerance in children, their role in the development of late and adult onset CD is less convincing. Nevertheless, in this study, we hypothesize that the intestinal microbial ecosystem as a whole rather than specific infections dictates the switch from tolerance to immune response in genetically susceptible individuals. However, at present, little is known about the potential role of the gut microbiota in CD. Compared to healthy individuals, adults or children with CD seem to be characterized by a somewhat different composition of the gut microbiota [20], [21], [22], [23], [24], [25]. Differences in microbial metabolites between fecal samples of CD patients and healthy controls point to a functional role of the microbiota in the pathogenesis of CD [22], [25], [26]. A significantly higher number of Gram-negative and potentially pro-inflammatory bacteria was found to be associated with the symptomatic presentation of CD [23]. The unbalanced microbiota in children with untreated CD seems only partially restored after long-term treatment with a gluten free diet [20], [24].

In the present study, we characterized the longitudinal changes from birth to 24 months of age in the microbial communities that colonize infants genetically at risk of CD and the impact of two different patterns of gluten introduction (early vs. late) on the gut microbiota, the fecal metabolome, and the switch from gluten tolerance to immune response, including onset of CD autoimmunity. For the first time, a combination of high-resolution culture-independent methods based on pyrosequencing of barcoded 16S rRNA gene amplicons, quantitative PCR, and metabolomic analysis was used to determine the composition and temporal changes of the gut microbiota as well as to identify potential metabolomic biomarkers associated with onset of CD in these at-risk infants. This study constitutes a definite proof-of-principle that these combined genomic approaches will be key to deciphering the role of the gut microbiota on CD onset.

## Results

### Subject enrollment

Forty-seven infants, first-degree relatives of patients with biopsy-proven CD, were enrolled before weaning (between birth and 6 months of age) (**Table S1**). It is noteworthy that all infants enrolled in this study were breastfed from birth to at least until 6 months of age (range 6 m–10 m, **Table S2**). A total of 34 infants were positive for HLA DQ2 and/or HLA DQ8 genotypes and fulfilled the inclusion criteria. From 6 to 12 months of age these infants were randomized to either a gluten-free diet (delayed exposure group A) or a gluten-containing diet (early exposure group B). At 12 months of age, they resumed a normal diet. A total of 13 infants in each group completed the protocol (for more details, see materials and methods section). Collected clinical data, including type of delivery, gestational age, weight, type of feeding and neonatal problems, are shown in **Table S1**.

For feasibility reasons, a subgroup of 8 infants in each group was randomly selected to perform microbiota and metabolome analysis. A total of 96 stool samples from these 16 babies were collected up to 24 months of age (**Table S2**). On average, 6 samples (range 3–8) were collected longitudinally from each subject spanning 8 different time points (7 and 30 days, 6, 8, 10, 12, 18 and 24 months).

### Celiac disease development and serologic evaluation

None of the eight babies in group A developed CD as defined by the appearance of CD anti-TTG antibodies, the onset of CD-related symptoms, and/or evidence of autoimmune enteropathy. One out of eight babies (12.5%) in group B developed CD at 24 months of age (AGA IgA 55.2 U/ml, AGA IgG 26.3 U/ml, anti-tTG IgG 51.9 U/ml, EMA IgA>1∶20) and started a gluten-free diet with subsequent normalization of serological tests at the follow up visit (outside of the clinical study).

**Figure 1:**
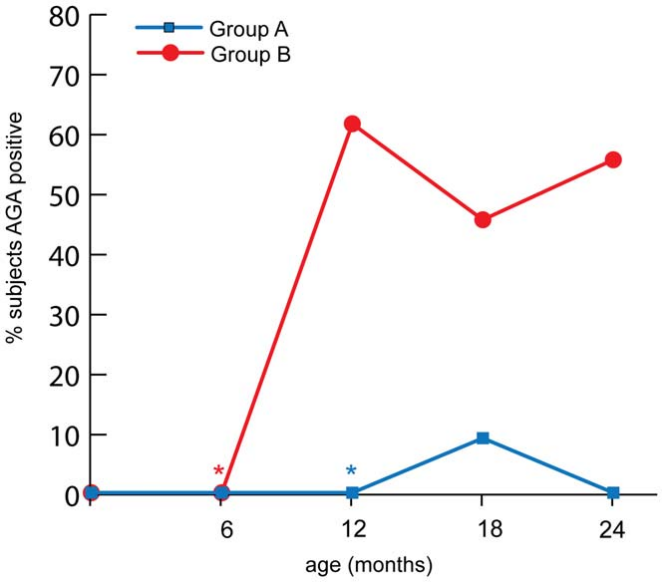
Cumulative incidence of AGA antibodies. Percent of AGA positive subjects enrolled in the study in each intervention group. * denotes time of gluten introduction, red, group B and blue, group A.

The comparison of AGA IgG positivity development over time in the two groups and its cumulative incidence are shown in **Table S3** and **Figure 1**. After normalizing the two groups by time of gluten exposure, the comparison shows a higher number of AGA IgG positivity in group B (**Figure 1**). These results suggest that early exposure to gluten in at-risk infants is associated with increased gut permeability to the protein in the intestinal lamina propria and a subsequent systemic immune response against gluten as testified by the IgG class of the AGA detected.

### Characterization of HLA DQ2^+^/DQ8^+^ infant gut microbiota from day 7 to 24 months of age

To characterize the succession of bacterial taxa that colonize the GI microbiota of DQ2^+^ and/or DQ8^+^ infants from 7 days to 24 months of age, we used pyrosequencing of barcoded 16S rRNA amplicons. Whole genomic DNA was extracted from each stool sample and the variable regions 1 and 2 (V1–V2) of the 16S rRNA gene were amplified using universal primers as described previously [27]. Using a Roche/454 FLX pyrosequencing instrument, we generated a dataset consisting of 394,002 high-quality, classifiable 16S rRNA gene sequence reads (average read length: 240 bp) with an average of 5,184 reads per samples.

**Figure 2:**
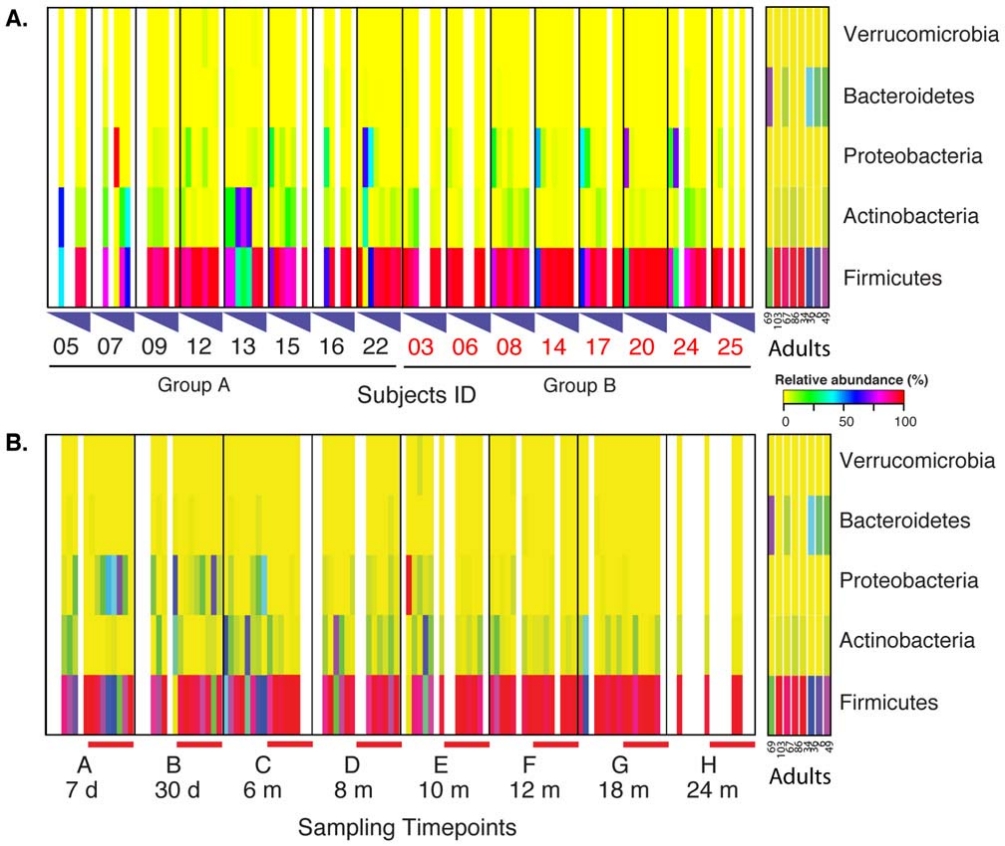
Heatmaps of relative abundance of bacterial phylum in the GI microbiota of samples collected longitudinally from 7 d to 24 months of age in DQ2^+^/DQ8^+^ infants (color key is indicated on the right). A. Samples are grouped by subjects ID and intervention groups. B. Samples are grouped by timepoints. Red bars indicate samples from subjects in intervention group B. Missing data point are indicated with a white vertical line. Stool samples collected from adult subjects that were processed in parallel are included for comparison (from left to right): subject 69: gluten-free diet for more than 24 weeks and HLA DQ2/8^+^; subject 103: HLA DQ2/8^+^, subject 67 HLA DQ2/8−; subject 86: diagnosed with CD and HLA DQ2/8^+^; subject 34: gluten-free diet for more than 24 months and HLA DQ2/8^+^; subject 36: HLA DQ2/8−; subject 6: HLA DQ2/8^+^; subject 49: HLA DQ2/8−.

**Figure 3:**
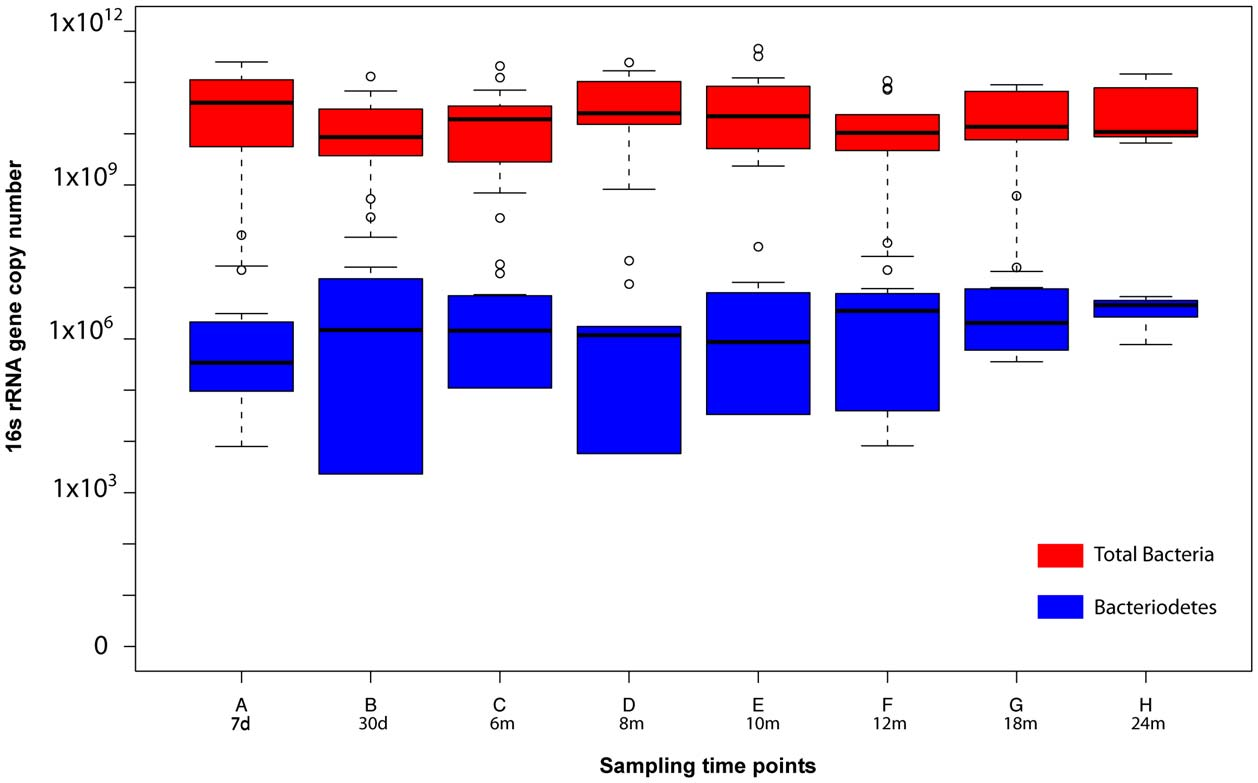
Quantitative real-time PCR of total 16S rRNA gene copies (red) and Bacteroidetes 16S gene copies (blue) for all samples analyzed at each time point in triplicate.

**Figure 4:**
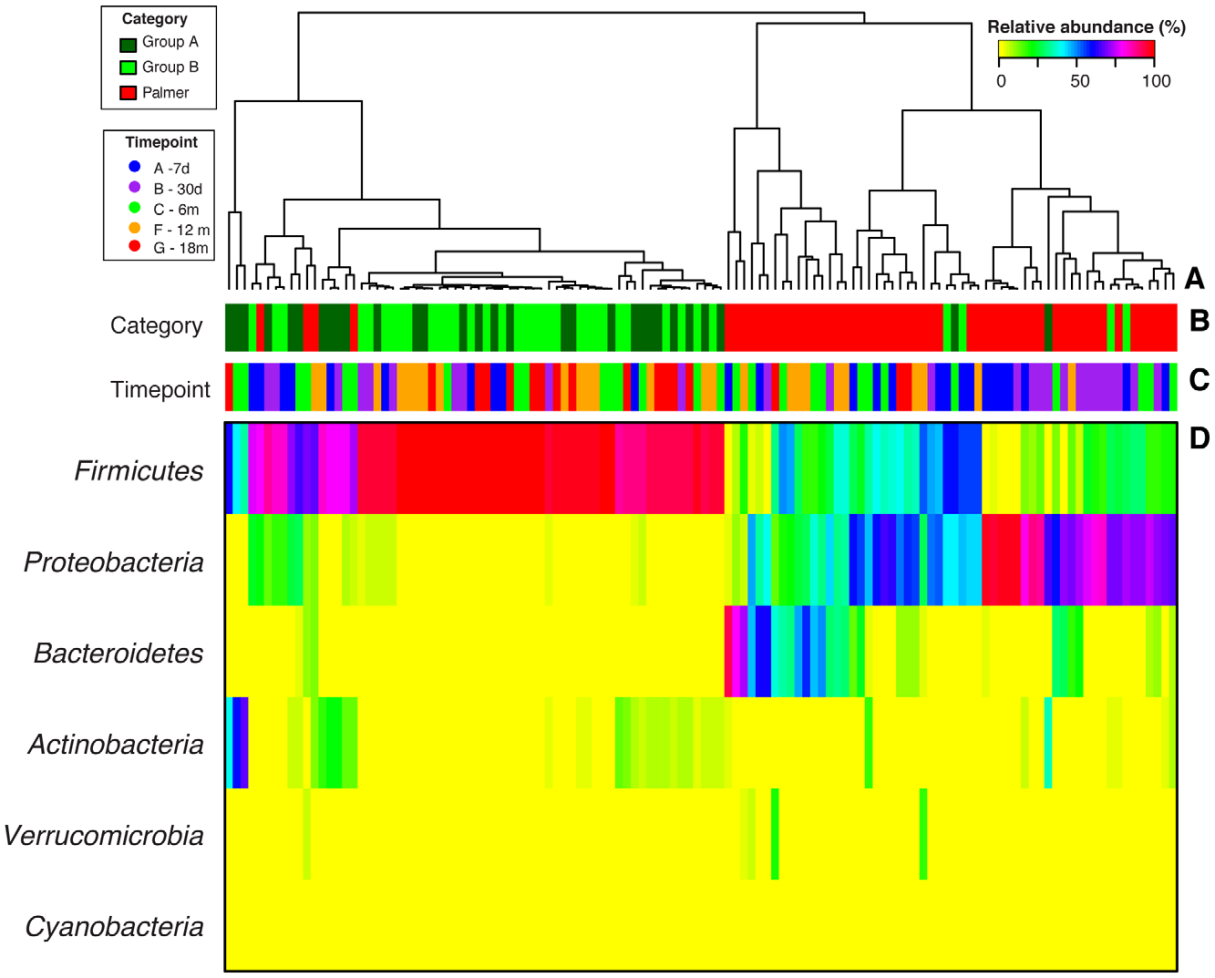
Heatmap of relative abundance of bacterial phylum of longitudinal samples from DQ2^+^/DQ8^+^ infants analyzed in this study and those of Palmer *et al.* [**28**] (D), Color keys are indicated on the upper right corner. A. Complete linkage clustering based on the phylum composition and abundance of GI microbiota. B. Color depicts the study and intervention group of the samples. C. Colors depict the time point at which the samples were collected. Time points D and E were omitted as no corresponding samples were collected in the Palmer *et al.* study [28].

Phylum level analysis of the colonization process revealed that at 7 days, most communities were comprised of a combination of members of the phyla Proteobacteria and Firmicutes, with Firmicutes dominating. Starting at 30 days of life, the abundance of Proteobacteria was diminishing while that of Actinobacteria was increasing. By 12 months the communities of both groups of infants were mainly composed of Firmicutes and Actinobacteria, while Proteobacteria represented less than 1% of the communities. At 18 months, Firmicutes were established as dominant members in more than 90% of the communities sampled (**Figure 2A and 2B**). Interestingly and contrary to previous reports from infants with uncharacterized genetic backgrounds [28], all communities of infants genetically at-risk for CD enrolled in this study were characterized by a low abundance of members of the phylum Bacteroidetes (undetectable to 1%). Quantitative real-time PCR indicated that the total number of 16S rRNA gene copies in the infants' GI communities ranged from 10^9^ to 10^10^ copies per gram of stool, while Bacteroidetes 16S rRNA gene copies ranged from 10^2^ to 10^7^ copies per gram of stool (**Figure 3**). This analysis confirmed that the relative abundance of Bacteroidetes was low, averaging three orders of magnitude lower than the total amount of bacteria, hence falling below the detection level of the sequencing analysis (represented by the depth of reads sampling, in this case, 5,184 reads per sample on average). A phylum level comparison with the study of Palmer *et al.* [28] who described the infant gut microbial colonization process in 14 infants up to 1½ years of age, is shown in **Figure 4**. Unlike in our findings, Palmer *et al*, demonstrated that Bacteroidetes is a common member of the infant GI microbiota after introduction of solid food at 6 months of age and thereafter (time point C, F and G).

The apparent transition and stabilization trends observed at the phylum level were not supported when analysis was performed at the genus level. Instability and high level of inter-individual variation were evident (**Figure S3**). The main genera observed in higher abundance belong to the phylum Firmicutes and were *Streptococcus*, *Lachnospira*, *Erysipetotrichae*, *Lactobacillus*, *Bryantella* and *Enterobacter*.

**Figure 5:**
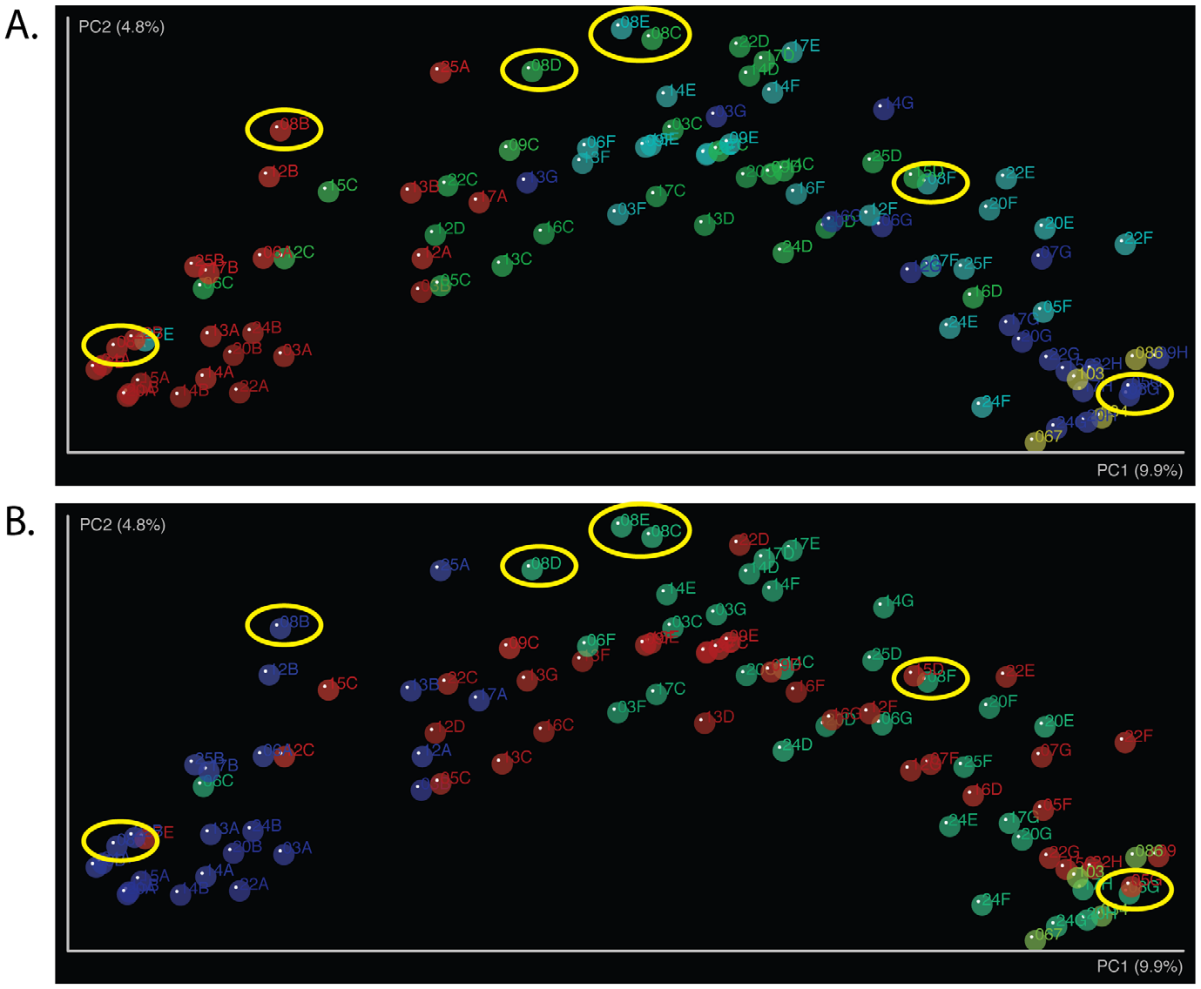
Principal Coordinate Analysis (PCoA) of unweighted UniFrac distances between samples. A. Samples are colored by time points, red, 7 d and 30 d, green, 6 m and 8 m, light blue, 10 m and 12 m, dark blue, 18 m and 24 m. B. Samples are colored by intervention groups. Group A: red, Group B: green. Blue: 7 d and 30 d time points prior to diet intervention. A and B: Gold, adult controls: subject 34 gluten-free diet for over 24 weeks and HLA DQ2/8^+^; subject 67 HLA DQ2/8−; subject 103 HLA DQ2/8^+^; subject 86; diagnosed with CD and HLA DQ2/8^+^.

Principal coordinate analysis (PCoA) of UniFrac distances reveals a high heterogeneity between community compositions for the first 30 days with communities spread along PC2 (**Figure 5A**, red spheres and **5B**, blue spheres). A directional pattern was observed in which communities converged over time toward higher level of similarities at 18 and 24 months along PC1 (**Figure 5**). At 24 months, GI communities, while not identical due to the lack of members of the phylum Bacteroidetes, tend to resemble those of adult subjects (**Figure 5** **, gold spheres**). In this analysis, different trends of convergence are observed for each intervention group (**Figure 5B**) after introduction of gluten. At 6 months, gluten introduction along with solid food tends to shift communities upward along PC2 (**Figure 5B**, green spheres) and PC1, while group A GI community compositions evolved moderately along PC1, with a few communities at 6 and 8 months still more similar to those found at 7 and 30 days (**Figure 5B**, red spheres). Using the Metastat software [29], we showed that the difference between group A and group B infants at time point C was statistically significant for the phyla Firmicutes (p-value = 0.00192) and Proteobacteria (p-value = 0.04588).

Subject 8 belonging to early gluten exposure group B was positive for AGA and anti-TTG/EMA at 18 and 24 months respectively and developed CD at 24 months. Interestingly, the GI microbiota composition of this subject appears to follow an outlier pattern as indicated on **Figure 5** (circled spheres) compared to the other DQ2^+^/DQ8^+^ infants. Introduction of gluten at 6 months of age in this subject triggered a major shift in community composition, which was comprised essentially of *Lactobacillus* until about 12 months of age (Figure S3A). Surprisingly, by 18 months of age, the GI microbial community of subject 8 appears more similar to GI microbiota of the other infants (**Figure 5**). Rarefaction curves were used to evaluate richness (i.e., number of unique bacterial taxa) in a sample (**Figure S4**). Interestingly, while most samples show an increase in richness over time (as shown by increasing slopes with time), subject 8 shows a dramatic reduction in bacterial richness in samples collected before the onset of CD (time points C, D and E corresponding to 6, 8 and 10 months of age).

### Fecal metabolites analysis

**Figure 6:**
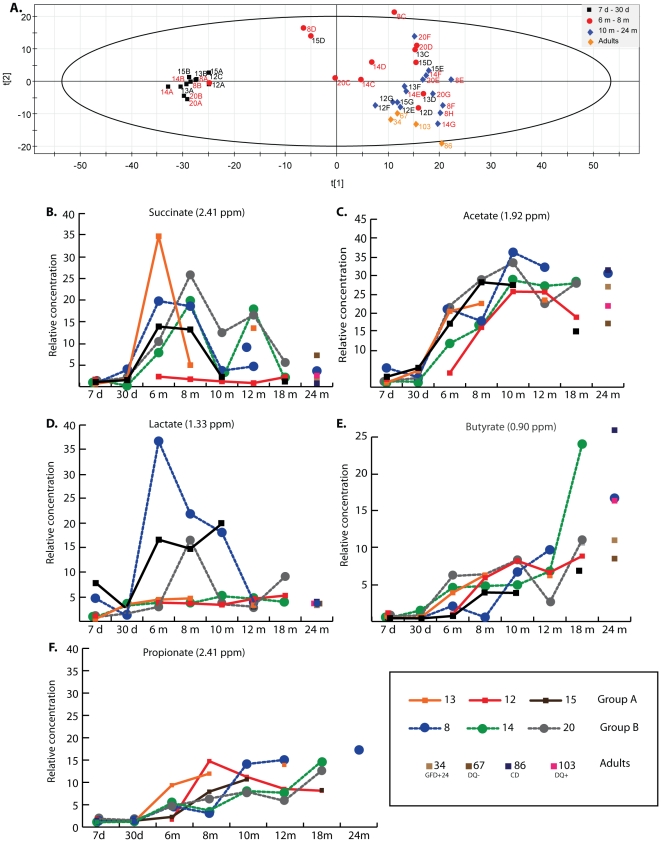
^1^H-NMR metabolomics analysis. A. PCA analysis of metabolomic profiles (A). Relative concentration of major metabolites measured from stool NMR profiles in selected subjects over the 24-month study period (B–F). B. Succinate; C. Acetate; D. Lactate; E. Butyrate and F. Propionate. The chemical shift (ppm) value of each metabolite is indicated above each panel.

A total of 17 fecal samples from three group A infants and 21 samples from three group B infants were processed for metabolic profiling by ^1^H NMR spectroscopy. These infants were selected to represent each intervention group, most time points and different outcomes (group B: subject 8: positive for CD autoantibodies and AGA/IgG; subject 14: negative for CD autoantibodies, but positive for AGA/IgG; subject 20: negative for CD autoantibodies and AGA/IgG). Overall, strong signals from lactose, glucose and other sugars (δ = 3.2–5.5 ppm) were observed in infants up to 30 days old. Resonances for acetate (δ = 1.92 ppm) and succinate (δ = 2.41 ppm) were somewhat pronounced as well. Starting at 6 months of age, the resonance levels of sugars dropped dramatically, while peaks for a number of amino acids and short chain fatty acids (SCFA) became more prominent. **Figure 6** shows the normalized concentration of selected metabolites present in stool samples from infants between 7 days to 24 months of age. Acetate levels stayed high during the whole time period and relative percentages increased after weaning, whereas sugars levels decreased (**Figure 6C**). Succinate levels initially increased after weaning and later dropped after the infants reached 10 months of age (**Figure 6B**). Butyrate was almost entirely absent in infants less than 6 months old and increased in most of the infants older than 10 months of age (**Figure 6E**).

Principal component analysis (PCA) was carried out on the normalized ^1^H NMR spectra of the fecal extracts to generate an overview of the variations between infants in intervention group A and B. Three principal components were calculated that accounted for 79% of the variance observed. While no clear separation of the metabolomes from group A and B was observed, the samples clustered by ages (**Figure 6A**). Using loading plot and NMR spectra analysis, we identified the metabolites responsible for the clustering (**Figure S5**). The 7-day and 30-day samples clustered together mainly because of high sugar contents, while the metabolomes of samples from infants older than 10 months clustered together mostly due to the presence of butyrate and propionate. Interestingly, the samples from 6- to 10-month old infants mostly clustered separately with higher levels of succinate. A few exceptions were noted, such as subject 20 (group B - 12 months sample (20F)), which clustered with samples from younger infants, and subjects 12 and 13 (group A – 8-month samples (12D and 13D)), which clustered with samples collected from older infants (**Figure 6A**). Those were the only differences observed between the two groups. Spectra for subject 8 (the only subject who developed CD at 24 months of age) at time points C (6 months) and D (8 months) were significantly different from those of the other infants at the same age. The loading plots indicated that the clustering in these samples was heavily influenced by higher lactate levels (**Figure S5**). The normalized lactate content was plotted against the age of the infants and is shown in **Figure 6D**. The lactate signals continued to be higher for subject 8 until 12 months of age before the first detection of positive antibodies. This period of high lactate signals (6–8 months of age) corresponds with a higher relative abundance of *Lactobacillus* spp., which dominated the stool microbial community of subject 8 with 88%, 57% and 81% at 6, 8 and 12 months, respectively.

## Discussion

We have demonstrated that infants genetically susceptible for CD (DQ2^+^/DQ8^+^/DQ2–8^+^) in which gluten introduction in their diet was delayed from 4–6 months of age to 12 months of age showed a decrease in immune response to gluten and a lower incidence of CD autoimmunity. Therefore, our results suggest that a delayed introduction of gluten in the diet of genetically susceptible infants can at least delay the onset of the disease. This observation was accomplished by using a prospective clinical study that included a dietary intervention with randomized and double-blind allocation to two diet groups (gluten-containing and gluten-free diets between the ages of 6 months to 1 year). This unique longitudinal study design allowed for the prospective collection of biological specimens that included stool samples, as well as behavioral and diet metadata.

In this study, we tested the effect of early exposure to gluten (6 months of age) or late exposure (12 months of age) on the immune response to gluten and development of CD autoimmunity in 26 genetically susceptible infants. We showed that delayed exposure to gluten has a positive effect on prolonging gluten tolerance and delaying onset of CD autoimmunity. During early childhood, antibodies can fluctuate between positive and negative, in line with the fluctuation seen in the incidence of AGA IgG in this study [30]. This study and the resulting observations are important because, despite the significant progress made in understanding the adaptive immunological aspects of CD pathogenesis, the early steps following intestinal mucosal exposure to gliadin that lead to the loss of tolerance and the development of the autoimmune process are still largely unknown. Increasing evidence in the literature seems to suggest a dysfunctional cross talk between innate and adaptive immunity as the key pathogenic element in the autoimmune process of the disease [31]. Recent retrospective studies also suggest that this dysfunctional cross talk could be influenced by the timing of gluten introduction into the diet, but more importantly, the studies suggest a potential role of the GI microbiota composition in subjects genetically susceptible to CD [32].

CD incidence is markedly increasing [13], [33], along with other immune-mediated disorders such as inflammatory bowel diseases (IBD), asthma or atopy [34]. The rapidity of the increase in disease rates could never be solely explained by changes in genetic make-up [35]. This hypothesis is supported by our recent prospective study on a single American cohort followed since 1974 indicating that CD autoimmunity in these subjects doubled between 1974 (1 of 501 subjects) and 1989 (1 of 219 subjects), thus excluding the genetic component as the cause of this increased prevalence [36]. Rather, alterations in host-commensal microbial interactions could have a pivotal role in the development of autoimmune disorders by triggering increased immune stimulation, epithelial dysfunction and enhanced mucosal permeability [37], [38]. The colonization of the GI tract, i.e. the succession of microbial communities that are established in the GI tract starting at birth, has been identified as a key factor influencing the risk of autoimmune and food-related diseases [39]. The involvement of intestinal colonization in the maturation of immune responses is well characterized in animal models, showing that both the intestinal-associated immune system and systemic immunity mature upon stimulation by the GI microbiota. Germ-free animals show extensive defects in the development of gut-associated lymphoid tissues and in antibody production, and have fewer and smaller Peyer's patches and mesenteric lymph nodes [40], [41]. Furthermore, it has been shown that germ-free animals have impaired development and maturation of isolated lymphoid follicles [42]. Recent studies also suggest that intestinal bacteria interact with the mammalian immune system to direct the differentiation of both pro- and anti-inflammatory T cell populations [43]. Because the microbiota has marked influences on the immune system, we have hypothesized that deviations from the “normal” development of the microbiota may alter the outcome of immune development and potentially predispose individuals to inflammatory diseases.

Our longitudinal study design allowed us to characterize the GI microbial colonization processes from 7 days to 24 months of age in DQ2^+^/DQ8^+^ infants from two intervention groups. One of the major finding of our study is that unlike in infants without a family history of CD and without genetic susceptibility to CD [28], the GI tract microbiota in DQ2^+^/DQ8^+^ infants appears to be lacking significant numbers of member of the phylum Bacteroidetes. The GI tract microbiota in these DQ2^+^/DQ8^+^ infants does not stabilize nor resembles adult microbiota at 1 year of age, and this characteristic remains at 24 months of age. A comparison with the study of Palmer et al. [28] highlights these major differences. In non-susceptible infants, the GI microbiota composition changes with different life stages, the most important of which is the introduction of solid food around 6 months of age. Overall the microbial ecosystem in each healthy baby achieves stability converging toward a profile more similar to that of an adult in the first year of life [28] with the level of Bacteroidetes ranging from a few percent to over 50% by 1 year of age. A recent paper by Koenig et al. [44] who described the GI colonization process in one infant for 2.5 years, further support the results of Palmer *et al.* [28]. In that study, Bacteroidetes comprised between 40 and 60% of the communities from 6 months to 2.5 years of age and the GI communities appeared to stabilize at 1 year of age [44]. It is evident that the colonization process in these infants with a higher abundance of Proteobacteria and Bacteroidetes throughout the first year of life and a lower abundance of Firmicutes, is dramatically different from those in the present study who are DQ2^+^/DQ8^+^ and with a family risk of CD. A complete clustering analysis of GI microbial communities from non-susceptible infants and the communities in this study revealed two major groups (**Figure 4**) separating infants by HLA genetic background and family history of CD. The result is highly influenced by the high abundance of Firmicutes and low abundance of Bacteroidetes in infants genetically susceptible for CD (mostly time points D, E, F and H [>6 months]), and by the high abundance of Proteobacteria in the earlier time points in the Palmer et al. study [28].

The ratio of Firmicutes over Bacteroidetes is known to vary throughout the lifespan. The ratio is lower in the first year of life, becomes higher in adulthood, and decreases in elderly [45]. Bacteroidetes have also been found to delineate profound differences between African and European children [46], with significantly higher levels of Bacteroidetes in African than European children. While certainly influenced by diet, a higher abundance of Bacteroidetes in African children appear to be protective against pathogens and other gastrointestinal diseases [46] and indicates a potential impact of the Western diet on the colonization and the establishment of the GI microbiota in Europe and other developed countries. The beneficial role of members of the phylum Bacteroidete*s*, including *Bacteriodes fragilis*, has been previously demonstrated [47], [48]. *B. fragilis* has been shown to establish a cross-talk between the GI microbiota and the intestinal epithelium [49]. *B. fragilis*, through the production of a polysaccharide capsule, directly induces the development of FoxP3+ regulatory T cells, which in turn produces anti-inflammatory cytokine directly in the gut [49]. The lack of Bacteroidetes could represent a major predisposing deficiency in infants genetically susceptible for CD, since it has been reported that quantitative and/or qualitative defects of FoxP3+ regulatory T cells affect immune tolerance surveillance and, therefore may lead to the autoimmune response typical of CD [50].

Our findings of decreased Bacteroidetes abundance in children at risk of CD are at odds with previous reports showing higher Bacteroidetes representation in CD children [23], [51]. However, substantial differences between our study design and the approach used by other investigators in regards to microbiota analysis (fluorescent in situ hybridization coupled with flow cytometry), age of children studied (older children), and clinical characteristics (children already affected by CD) make previous findings not directly comparable to our results. Indeed, to our knowledge, our study is the first prospective report on infants at risk of CD that analyzes the microbiota dynamic over time starting from birth, using state-of-the-art microbiome 16S rRNA short amplicon pyrosequencing combined with quantitative PCR and ^1^H NMR spectroscopy. The key finding of our report is the lack of maturation of the gut microbiota within the first 2 years of life in infants at risk of CD characterized by a relative absence of Bacteroidetes and a parallel high abundance of Firmicutes. It is important to note that differences in stool sampling and storage, DNA extraction and purification methods, 16S rRNA gene PCR primer pairs, and sequencing methods between all studies, including this and the Palmer *et al.* study [28] could potentially explain some of the variations observed in the composition of the GI microbial community. However, it is unlikely that the DNA extraction method used in this study is responsible for the observed low abundance of Bacteroidetes, as high abundance of this phylum was detected in stool samples (Figure 2) from adult subjects (HLA DQ2/8- and some HLA DQ2/8^+^ on gluten free diet for more than 24 months). Furthermore, the method is commonly used to extract stool samples in our laboratory and members of the phylum Bacteroidetes and other phyla are often identified (data not shown).

Metabolomic profiling of selected subjects and samples revealed that few metabolites appeared important and accompanied the succession of the microbial taxa colonizing the GI during the first two year of life. The GI metabolome during the first 6 months of life reflects the infants' diet of exclusively milk comprised mainly of polysaccharides and other sugars. This unique metabolic profile is very similar in all infants. However, once solid food is introduced at 6 months of age, a major shift occurs and the SCFA succinate, acetate, propionate and butyrate are found in the feces. By 24 months, butyrate and acetate are the main SCFA present in the metabolome of these infants. A recent study found Bacteroidetes associated with the presence of butyrate, and acetate and most strongly with propionate, while Firmicutes was negatively associated with these SCFAs [44]. The role of these SCFAs is thought to be protective and the results of the breakdown of complex plant polysaccharides by Bacteroidetes [52]. Propionate was not found in high level in the feces of the infants studied compared to other published studies [44]. This finding correlates with the concordant lack of Bacteroidetes in the GI microbiota of infants genetically susceptible for CD. One can envision that the high abundance of Firmicutes and the low abundance of Bacteroidetes in these infants results in lower levels of SCFAs in the GI tract and a diminished GI health and/or a predisposition for CD or other autoimmune diseases. More quantitative methods and a higher number of subjects would be needed to confirm this finding. The metabolic profiles are consistent across the infants and cluster by age (**Figure 6A**). This finding supported the concept that while GI microbiota may differ in microbial species composition and abundance, they conserve a functional core, whether it is as conserved gene content [53] or as observed in this study in conserved metabolic output. Our metabolomic analysis did not reveal any consistent differences between infants from each dietary intervention group (**Figure 6**). However, because one of the infants (subject 8) was diagnosed with CD at 24 months, we had the opportunity to prospectively examine both the GI microbiota colonization process and its associated metabolome. Interestingly, between 6 and 12 months of age, the metabolome of subject 8 contained high levels of lactate (**Figure 6D**), which correlated with the presence of high levels of *Lactobacillus* spp. in the GI microbiota (**Figure S3**). Two more subjects showed elevation of lactate in their metabolome profile. Subject 15 (group A) showed elevated and sustained lactate levels within an interval period similar to subject 8 (from 6 to 10 month of age) (**Figure 6D**). Interestingly, subject 15 developed type 1 diabetes (T1D), another autoimmune disease, at 22 months. The second subject with elevated lactate (subject 20, group B) showed only a very transient increase in lactate at age 8 months which returned to baseline levels similar to other infants by 10 months of age (**Figure 6D**). Based on these results, it is tantalizing to hypothesize that a decrease in *Lactobacillus* spp., with a subsequent decrease in lactate production during a crucial time of maturation of mucosal immunity functions (between 6 and 12 months of age) can leads to loss of tolerance to non-self antigens (gluten in case of CD, unknown antigen(s) in case of T1D) in genetically susceptible individuals. Larger studies with more cases are necessary to support this hypothesis.

Subject 8 also experienced reduced bacterial richness during the intervention period compared to the other infants, which showed increased richness with time (**Figure S4**). While this was observed in only one infant, it introduces the possibility of discovering potential biomarkers that could be predictive to the development of autoimmunity in CD; a longitudinal study design is essential to achieve this goal.

In summary, infants genetically susceptible for CD may benefit from delayed exposure to gluten from 6 months of age to at least 12 months of age. While the molecular mechanisms underlying the benefit are yet unknown, it might be related to a lack of maturity of the GI microbiota in these infants, and we hypothesize that the introduction of gluten in an immature GI microbiota could trigger or accelerate the development of autoimmunity. By combining 16S rRNA gene short amplicon pyrosequencing, quantitative PCR and ^1^H NMR spectroscopy to analyze the microbiota of infants with genetic predisposition for CD over the first two years of life, we have characterized the GI colonization process and its metabolic output in infants genetically susceptible for CD. The data presented here, while derived from a relatively small number of subjects, suggest significant differences between the developing microbiota of infants with a genetic predisposition for CD and those from infants with a non-selected genetic background. Furthermore, the metabolic output of the GI microbiota in these infants while similar to one another within age groups might reflect a potential dysbiosis of the GI microbiota and lead to less than optimal cross-talk with the host to promote health. Interestingly, one of the infants in the study was diagnosed with CD at 24 months of age. The retrospective analyses of the GI microbiota and metabolomic data suggest that potential specific biomarkers might be identified that would be predictive for autoimmune development in subjects genetically at risk, possibly leading to the development of potential interventions during the pre-clinical phase of the disease to arrest the loss of tolerance to gluten and, therefore, to prevent the onset of CD autoimmunity.

## Materials and Methods

### Study design and sample collection

The study was prospective and included a dietary intervention with randomized and double-blind allocation to two diet groups. Infants with first-degree relatives diagnosed with biopsy-proven CD were enrolled between 2005 and 2009 (**Figure S1**). The HLA-DQ2/DQ8 determination was performed soon after birth, when possible on cord blood, or at the time of recruitment. Positive subjects for HLA-DQ2 and/or DQ8 genotype were included in the interventional study. Data on clinical and dietary history were recorded including type of delivery, gestational age, birth weight, type of feeding, antibiotic use and neonatal complications. All infants received exclusive milk feeding during the first 6 months of life. From weaning (6 months of age) to 12 months all recruited infants were on the same basic gluten-free diet and were randomly assigned to two different arms. In group A, infants received a daily supplement consisting of purified corn starch (3 g from age 6–9 months and 5 g from age 9–12 months), while group B infants received a daily supplement consisting of purified gluten from hexaploid wheat (3 g from age 6–9 months and 5 g from age 9–12 months). After 12 months of age all children (group A and B) were allowed an appropriate unrestricted diet and were followed every 6 months up to 24 months. From weaning to 12 months, the clinical data, the adherence to the dietary protocol, and the amount of intervention food supplement ingested were followed and recorded. CD serology (anti-gliadin (AGA) IgA and IgG antibodies, anti-tissue transglutaminase (tTG) IgA and IgG antibodies, anti-endomysial (EMA) IgA antibodies) and total IgA measurement were performed at the time of recruitment and at each 6-month follow up visit. Stool samples were collected at 7 and 30 days and at6, 8, 10, 12, 18 and 24 months of age. Infant stool samples were obtained by the parents using stool collection vials and were immediately stored in home freezers at −20°C and then transported frozen to the laboratory within 24 h where the samples were stored at −80°C until processed.

The University of Maryland School of Medicine Institutional Review Board approved the study protocol and written informed consent was obtained from the parents of all children enrolled.

### Subject enrollment and retention

A total of 47 at-risk infants were screened. After HLA typing, 34 (72%) infants were positive for HLA-DQ2 and/or DQ8 genotype and, therefore, met the enrolling criteria. The families of 4 of these infants refused randomization. The remaining 30 infants were blindly randomized to the two groups once they reached weaning age (**Figure S1**). Since this was an ongoing enrolling study, more patients were enrolled in group B (early exposure to gluten, n = 17) than in group A (late exposure to gluten, n = 13) to counterbalance for non-compliance to the feeding intervention (n = 3) or loss at follow up (n = 1) of 4 infants assigned to group B (**Figure S2**).

### HLA typing

The HLA II alleles (DQA1*0201, DQA1*03, DQA1*05, DQB1*02, DQB1*0302, DRB1*03, DRB1*04 and DRB1*07) were typed using the DiaGene kit (Palermo, Italy) at the University of Maryland School of Medicine, Center for Celiac Research. PCR amplicons were resolved by electrophoresis on 2% agarose gel and stained using ethidium bromide.

### CD serology

Both anti-tTG and anti-AGA antibodies were determined by ELISA assay using an ImmunoCAP 100 instrument (Phadia, Portage MI) and as recommended by the manufacturer. Values higher than 7.0 AU were considered positive for all assays. EMA was detected by indirect immunofluorescence assays (Scimedx, Denville, NJ) using monkey esophagus as substrate and as recommended by the manufacturer. Values above 1∶10 were considered positive.

### Definition of gliadin immune response and CD autoimmunity

Gliadin immune response was defined as the presence of IgG and/or IgA anti-AGA antibodies above the 7.0 AU. CD autoimmunity was defined as the presence of tTG antibodies higher than 7 AU and subsequent positivity to EMA, with or without AGA positivity.

### Total DNA extraction from stool

Frozen fecal material (150 mg) was mixed with 1 ml of 0.05 M potassium phosphate and transferred to a FastPrep Lysing Matrix B tube (Bio 101). Cell lysis was initiated by adding 5 µl lyzozyme (10 mg/ml), 15 µl of mutanolysin (11,700 U/ml; Sigma-Aldrich, St. Louis, MO) and 5 µl of lysostaphin (4,000 U/ml in sodium acetate; Sigma-Aldrich). Following a 30 min incubation at 37°C, 10 µl proteinase K (20 mg/ml), 50 µl 10% SDS, and 2 µl RNase A (10 mg/ml) were added to the mixture and incubated for 45 min at 55°C. Microbial cells were further lysed by mechanical disruption using a bead beater (FastPrep instrument, Qbiogene, Montreal) set at 6.0 m/s for 40 sec. The lysate was processed using the ZYMO Fecal DNA extraction kit (ZYMO Research, Irvine, CA) omitting the lysis steps and according to the manufacture's recommendation. The samples were eluted with 100 µl of hot molecular biology-grade water (56°C) and quantified using Picogreen.

### Pyrosequencing of barcoded 16S rRNA gene amplicons

The two universal primers 27F and 338R were used for PCR amplification of the V1–V2 hypervariable regions of the 16S rRNA gene [54]. The 338R primer included a unique sequence tag to barcode each sample. The primers were as follows: 27F - 5′- GCCTTGCCAGCCCGCTCAGTC**AGAGTTTGATCCTGGCTCAG**-3′ and 338R - 5′- GCCTCCCTCGCGCCATCAGNNNNNNNNCA**TGCTGCCTCCCGTAGGAGT**-3′, where the underlined sequences are the 454 Life Sciences® FLX (454 Life Sciences, Branford, CT) sequencing primers B and A in 27F and 338R, respectively, and the bold font denotes the universal 16S rRNA primers 27F and 338R. The 8-bp barcode within primer 338R is denoted by 8 Ns. 16S rRNA genes were amplified in 96 well microtiter plates using AmpliTaq Gold DNA polymerase (Applied Biosystems, Carlsbad, CA), and 50 ng of template DNA in a total reaction volume of 50 µl. Reactions were run in a PTC-100 thermal controller (MJ Research, Hatboro, PA) using the following cycling parameters: 5 min of denaturation at 95°C, followed by 20 cycles of 30 sec at 95°C (denaturing), 30 sec at 56°C (annealing) and 90 sec at 72°C (elongation), with a final extension at 72°C for 7 minutes. Negative controls without a template were included for each barcoded primer pairs. The presence of amplicons was confirmed by gel electrophoresis on a 2% agarose gel and staining with SYBRGreen. PCR products were quantified using a GelDoc quantification system (BioRad, Hercules, CA) and equimolar amounts (∼100 ng) of the PCR amplicons (96 samples) were mixed in a single tube. Amplification primers and reaction buffer were removed by processing the amplicons mixture with the AMPure Kit (Agencourt, Beverly, MA). The purified amplicon mixtures were sequenced by 454 FLX pyrosequencing using 454 Life Sciences® primer A by the Genomics Resource Center at the Institute for Genome Sciences, University of Maryland School of Medicine using protocols recommended by the manufacturer and as amended by the Center.

Sequences were binned by samples using the sample-specific barcode sequences and trimmed (removal of the barcode and primer sequences). We used criteria previously described to assess the quality of sequence reads. Briefly, to pass, a sequence read had to (a) include a perfect match to the sequence tag (barcode) and the 16S rRNA gene primer; (b) be at least 200 bp in length; (c) have no undetermined bases; and (d) have at least a 60% match to a previously determined 16S rRNA gene sequence. Phylum and genus level taxonomic assignments were performed using the RDP Classifier [55]. For the RDP classifier, we required >50% confidence for all calls. Trimmed pyrosequence reads were processed with the CloVR-16S pipeline [56] of the Cloud Virtual Resource (CloVR) [57]. CloVR-16S integrates component of the QIIME [58] and Mothur [59] packages to align reads with Pynast [60], construct phylogenetic trees with FastTree2 [61], calculate UniFrac distances, and generate PCoA plots based on UniFrac distances. OTUs were computed using the pick_otus and pick_rep_set workflow scripts in QIIME. High-quality sequences were first clustered into OTUs using UCLUST [62] with a 97% identity threshold. The most abundant sequence in each OTU was then selected as its representative member. Rarefaction curves were calculated using Mothur with OTUs generated by QIIME/UCLUST as input.

Sequences have been deposited in the Short Read Archive under accession number ###### (pending).

### Quantitative PCR assay

A separate real-time quantitative PCR (qPCR) assay was used to amplify and quantify rRNA gene copy number from total bacteria and those belonging to the phylum Bacteroidetes. Total pan-bacterial 16S rRNA gene qPCR was performed using primers 5′-CCTACGGGDGGCWGC-3′, ‘5-GGACTACHVGGGTMTCTAATC-3′ as forward and reverse primers respectively and 6FAM-CAGCAGCCGCGGTA-MGBNFQ as TaqMan probe. Bacteroidetes qPCR were performed using primers 5′-AACGCTAGCTACAGGCTTAACA-3′, 5′-ACGCTACTTGGCTGGTTCA-3′, and 6FAM-CAATATTCCTCACTGCTGCCTCCCGTA-TAMRA, for the forward, reverse and probe primers respectively [63]. For both qPCR assays, each 20-µl reaction contained 1X Invitrogen (Grand Island, NY) qPCR SuperMix, 1.8 µM of each primer, 0.2 µM of the probe, and 1 µl of extracted DNA. The thermal cycling program consisted of 50°C for 3 min for uracil-*N*-glycosylase activation, 95°C for 5 min for denaturation, followed by 45 cycles of 95°C for 30 s, 55°C for 30 s and 60°C for 1 min. Reactions were carried out in a Prism 7900HT Sequence Detection System (Applied Biosystems). Ten-fold serial dilutions of known quantities of cloned 16S rRNA genes and Bacteroidetes targets were used to generate standard curves (ranging from 10^1^ to 10^8^ gene copies/µl). Absolute 16S rRNA genes abundance was calculated based on the standard curves using SDS software version 2.1 (Applied Biosystems). The total copy number of 16S rRNA genes was normalized to gram of stool sample processed. All of the reaction mixtures were run in triplicate and each plate included three no-template controls.

### Comparative analysis with previous studies datasets

The study by Palmer et al. [28] used a microarray platform to evaluate the present/absence and abundance of microbial taxa. The relative proportion of each phylum in the datasets was calculated by normalizing the raw intensity values for each phylum to the total amount of intensity for all phyla. This procedure was performed for each samples and provided the relative percent abundance of phyla in each samples.

### ^1^H-Nuclear Magnetic Resonance Spectroscopy (NMR) spectroscopy and metabolomic analysis of fecal samples

For each sample, 100 mg of frozen fecal sample was placed into a 1.5 ml centrifuge tube and 1.0 ml of deuterated water (D_2_O) added as extraction solvent. The sample was homogenized by vortex mixing for 1 min and stored on ice for 10 min. After centrifugation (10 min, 13,000 rpm at 4°C), the supernatant was collected and filtered through a 0.45 µm MCE filter (Fisherbrand, Pittsburg, PA) to remove particulates. The filtered fecal solutions were stored at −80°C prior to analysis. As a control to determine whether any contamination may have been introduced by filtration, a sample containing only deuterated water was also filtered using the same procedure.

NMR was used to monitor the metabolite profiles. Two hundred µl of the resulting fecal extract was diluted with D_2_O yielding a 500 µl sample containing 50 mM phosphate buffer at pH 7.0 and 30 µM sodium 3-(trimethylsilyl) propionate -2,2,3,3-d4 as internal chemical shift reference. The resulting solution was mixed by vortex and then centrifuged at 13,000 rpm for 2 min and transferred to a 5 mm NMR tube. All ^1^H-NMR experiments were carried out at 25°C on a Varian AS500 spectrometer operating at a proton NMR frequency of 499.75 MHz. One-dimensional spectra were recorded using standard tnnoesy pulse sequence. Each spectrum consisted of 128 transients with a spectral width of 12 ppm and relaxation delay of 5.0 s. All free induction decays were Fourier transformed with an exponential function equivalent to a 0.3 Hz line-broadening factor and the spectra were zero filled to 32K points. The resulting spectra were manually phased and baseline corrected using ACDLABS (version 10.0, Advanced Chemistry Development, Inc., Toronto, ON, Canada). For ^1^H-NMR signal assignment purposes, two-dimensional (2-D) J-resolved spectroscopy [64] and total correlation spectroscopy (TOCSY) [65] NMR spectra were acquired for two selected samples. J-resolved spectra were collected using 128 scans per 32 increments with 5000 Hz spectral width in F2 and 36 Hz in F1. The TOCSY spectra were recorded with a data matrix of 2048×128 with spectral width of 5000 Hz in F2 and F1. Sixty-four scans were acquired and a mixing time of 80 ms was used. All 2-D NMR data were processed with the software package NMRPipe [66].

For PCA analysis, the ^1^H-NMR spectra were reduced to 119 integrated regions of varied width corresponding to the region of δ = 0.40–8.50 ppm using intelligent bucketing from ACD Labs (Toronto, ON, Canada). The region from δ = 4.7–5.0 ppm was excluded from the analysis to avoid the phasing effects of the pre-saturation of the residual water signal. Normalization of the integrals to the total sum of the spectrum was carried out on the data prior to principle component analysis (PCA) to allow for differences in signal-to-noise. PCA was performed with the SIMCA-P software (version 10.0, Umetrics, Sweden). The data were pre-processed by Pareto scaling.

The relative concentration of metabolites was represented by the integral values of their peak regions. To make the individual peak areas comparable between samples, normalization of the total integral sum of the whole spectrum (set as 1000) was carried out. The values were then divided by the number of protons of the corresponding peak. **Figure 6** shows the relative concentration of selected metabolites present in the samples obtained from infants age 7 days to 24 months.

## Supporting Information

Figure S1 Schematics of the clinical study design. GFD: Gluten-Free Diet.
(TIF)

Figure S2 Flow-chart depicting infants enrolled in the study and those that were selected for microbiota characterization.
(TIF)

Figure S3 Heatmaps of relative abundance of bacterial genera in the GI microbiota of samples collected longitudinally from 7 d to 24 months of age in DQ2^+^/DQ8^+^ infants (color key is indicated on the right). A. Samples are grouped by subjects ID and intervention groups. B. Samples are grouped by time points. Red bars indicate samples from subjects in intervention group B. Taxa are ordered from most abundant to least abundant. “Unclassifiable reads” represent a set of reads for which statistical support was not achieved by the RDP classifier to be assigned to a specific genus.
(TIF)

Figure S4 OTU-level rarefaction curves for each subject and time point. Infants in group B are indicated by a red bar. Time points are indicated by different colored lines.
(TIF)

Figure S5 Loading scatter plot associated with the PCA metabolomics profiles analysis presented in Figure 6A. Metabolites that influence the PCA scattering are indicated and colored accordingly. The confidence ellipse is shown as calculated by SIMCA-P. Observations situated outside the ellipse are considered outliers.
(TIF)

Table S1 Characteristics of the study subjects.
(PDF)

Table S2 Stool samples collected from each subject.
(PDF)

Table S3 Antibody positivity cumulative incidence.
(PDF)
