# Supplementary figures and images for: Proof of Concept of Microbiome-Metabolome Analysis and Delayed Gluten Exposure on Celiac Disease Autoimmunity in Genetically At-Risk Infants
Source: PLoS One. 2012 Mar 14;7(3):e33387. doi: 10.1371/journal.pone.0033387 (PMC3303818; doi:10.1371/journal.pone.0033387)

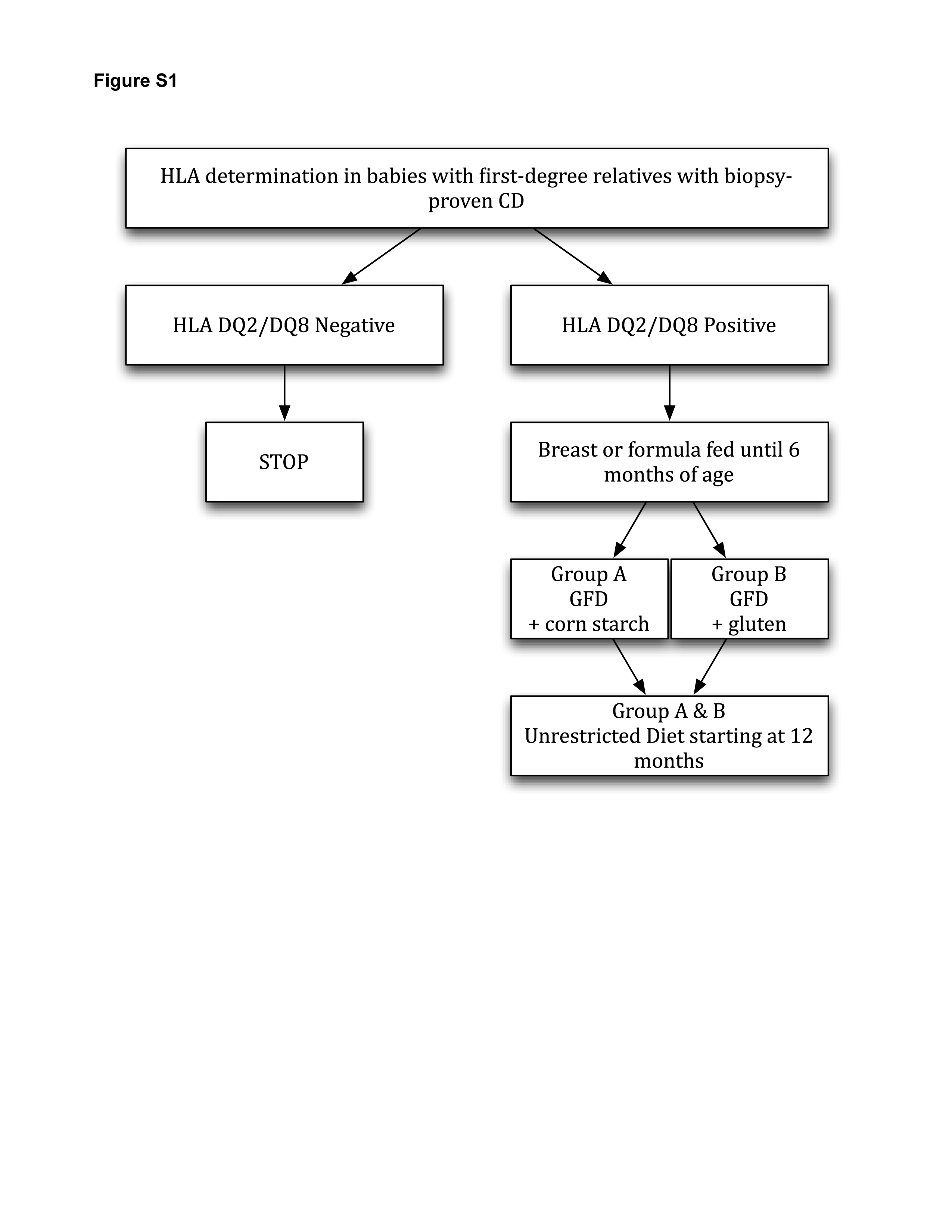

Supplement: Figure S1 — Schematics of the clinical study design. GFD: Gluten-Free Diet. (TIF) [file pone.0033387.s001.tif]

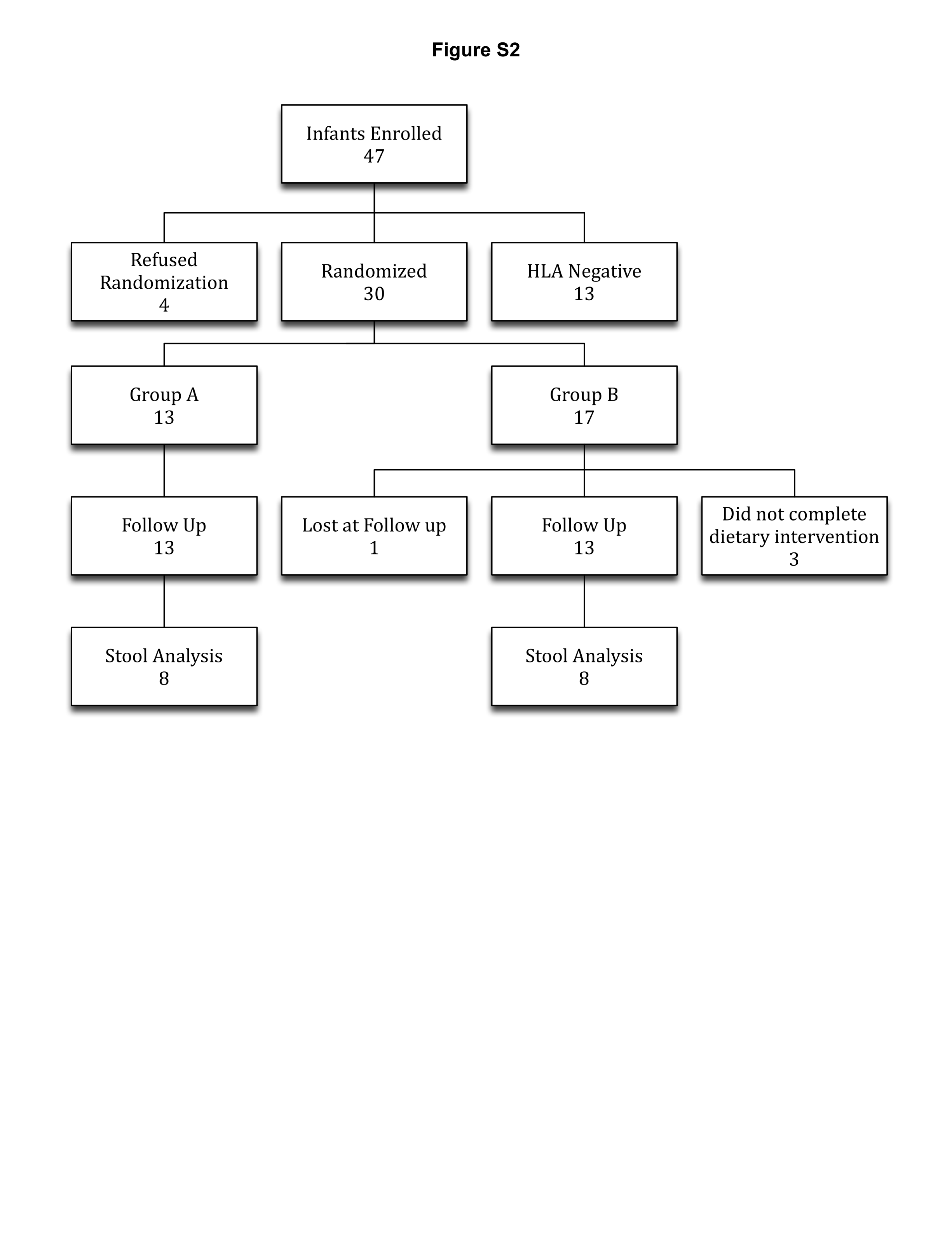

Supplement: Figure S2 — Flow-chart depicting infants enrolled in the study and those that were selected for microbiota characterization. (TIF) [file pone.0033387.s002.tif]

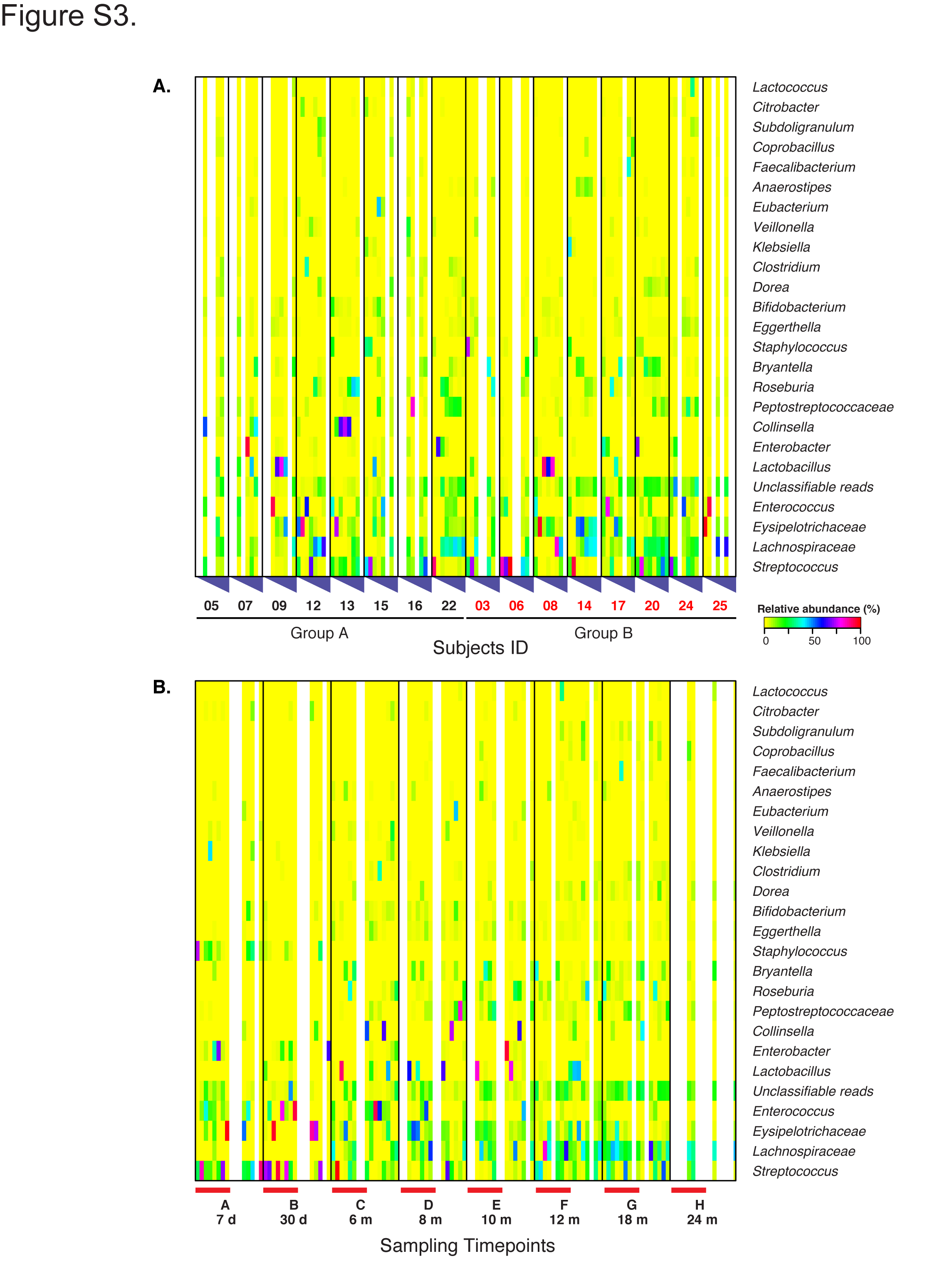

Supplement: Figure S3 — Heatmaps of relative abundance of bacterial genera in the GI microbiota of samples collected longitudinally from 7 d to 24 months of age in DQ2+/DQ8+ infants (color key is indicated on the right). A. Samples are grouped by subjects ID and intervention groups. B. Samples are grouped by time points. Red bars indicate samples from subjects in intervention group B. Taxa are ordered from most abundant to least abundant. “Unclassifiable reads” represent a set of reads for which statistical support was not achieved by the RDP classifier to be assigned to a specific genus. (TIF) [file pone.0033387.s003.tif]

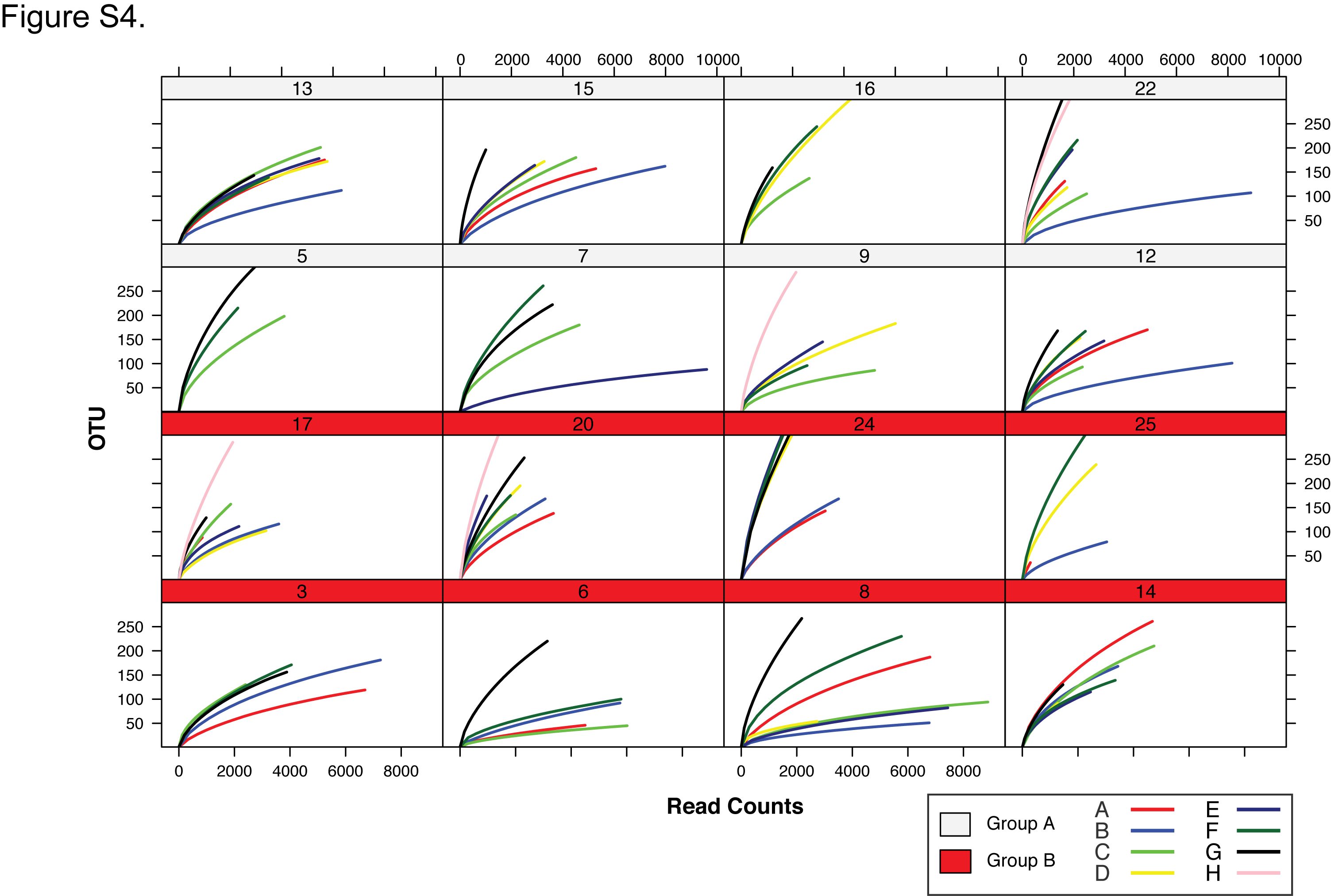

Supplement: Figure S4 — OTU-level rarefaction curves for each subject and time point. Infants in group B are indicated by a red bar. Time points are indicated by different colored lines. (TIF) [file pone.0033387.s004.tif]

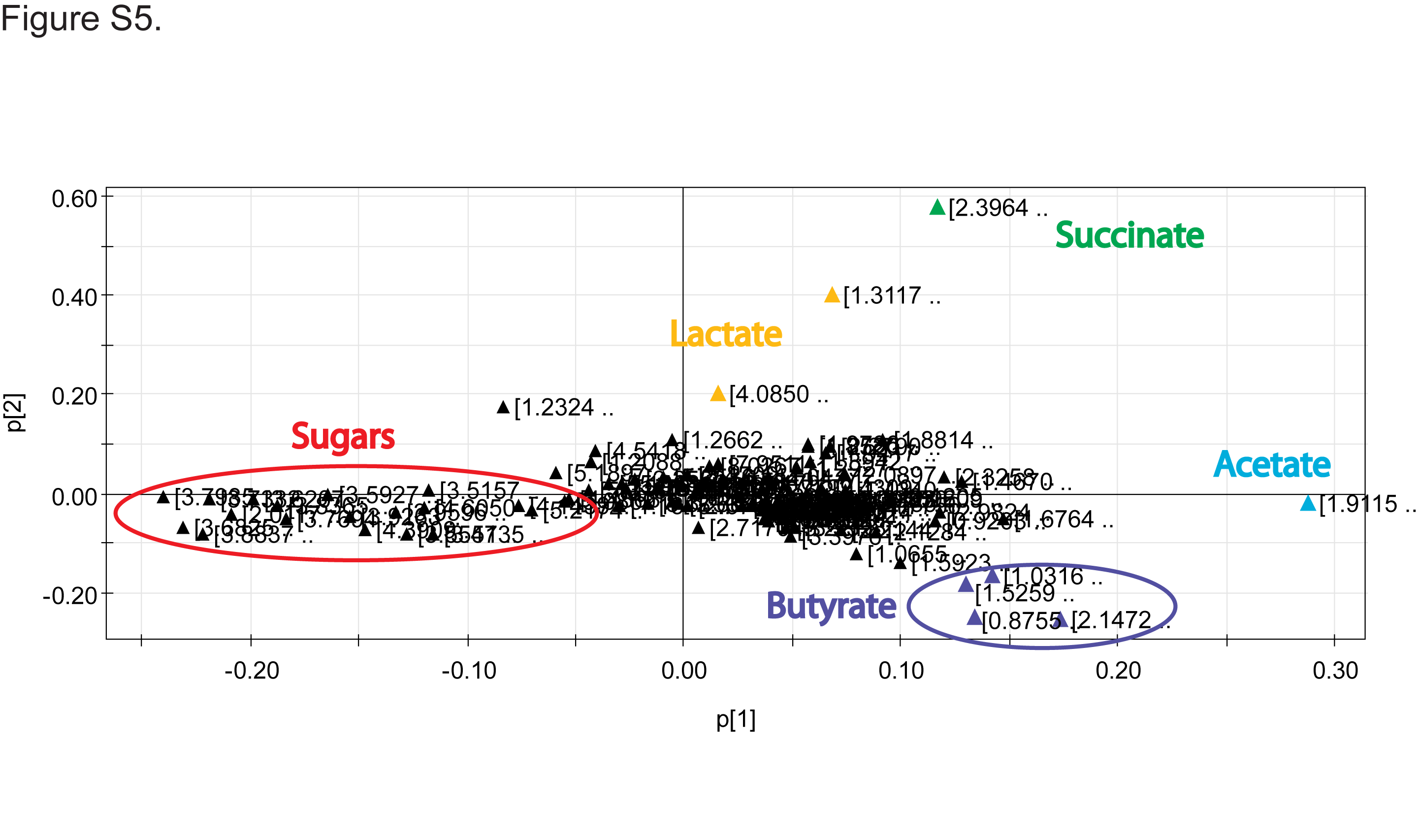

Supplement: Figure S5 — Loading scatter plot associated with the PCA metabolomics profiles analysis presented in Figure 6A. Metabolites that influence the PCA scattering are indicated and colored accordingly. The confidence ellipse is shown as calculated by SIMCA-P. Observations situated outside the ellipse are considered outliers. (TIF) [file pone.0033387.s005.tif]
