# Supplementary material for: Proof of Concept of Microbiome-Metabolome Analysis and Delayed Gluten Exposure on Celiac Disease Autoimmunity in Genetically At-Risk Infants
Source: PLoS One. 2012 Mar 14;7(3):e33387. doi: 10.1371/journal.pone.0033387 (PMC3303818; doi:10.1371/journal.pone.0033387)
Supplement: Table S1 — Characteristics of the study subjects. (PDF) [file pone.0033387.s006.pdf]

Table S1. Characteristics of the study subjects

	Group A	Group B
**Gestational Age (weeks)**	39.5 (38.8)	39.4 (39)
**Vaginal Delivery**	13/13 (8/8)	11/13+ (8/8)
**Birth Weight (kg)**	3.2 (3.4)	3.4 (3.4)
**Caucasian**	13/13 (8/8)	13/13 (8/8)
**Sex (M:F)**	6:7 (4:4)	7:6 (4:4)
**Neonatal Jaundice**	3/13 (3/8)	4/13 (4/8)
**Exclusive Breast Feeding***	4/13x (4/8+)	7/13% (4/8#)
**HLA DQ2:DQ8:DQ2/8**	10:2:1 (5:2:1)	9:4:0 (5:3:0)

*** exclusivly breast feeding until 6 months of age**

+ 1 subject has no information

x 2 subjects do not have information

% 3 subjects do not have information

4 subjects do not have information

stool analysis samples in parenthesis

Table S2. Stool samples collected from each subjects

ID	Group	**A** 7d	**B** 30d	**C** 6m	**D** 8m	**E** 10m	**F** 12m	**G** 18m	**H** 24m
5	A			5C			5F	5G	
7	A			7C		7E	7F	7G	
9	A			9C	9D	9E	9F		9H
12	A	12A	12B	12C	12D	12E	12F	12G	
13	A	13A	13B	13C	13D	13E	13F	13G	
15	A	15A	15B	15C	15D	15E	15F	15G	
16	A			16C	16D		16F	16G	
22	A	22A	22B	22C	22D	22E	22F	22G	22H
6	B	6A	6B	6C			6F	6G	
3	B	3A	3B	3C			3F	3G	
8	B	8A	8B	8C	8D	8E	8F	8G	
14	B	14A	14B	14C	14D	14E	14F	14G	
17	B	17A	17B	17C	17D	17E		17G	17H
20	B	20A	20B	20C	20D	20E	20F	20G	20H
24	B	24A	24B	24C	24D	24E	24F	24G	
25	B	25A	25B		25D		25F		

Table S3. Antibody positivity cumulative incidence

Exposure to Gluten	Group A	Group B
	#/screened (%) Ab positive subjects	#/screened (%) Ab positive subjects
6m	0/13 (0%)	0/13 (0%)
12m	0/12 (0%)[a]	8/13 (61.5%)
18m	1/11 (9.1%)[a]	5/11 (45.5%)[a]
24m	0/10 (0%)[a]	5/9 (55.5%)[a,b]

[a], not all 13 subjects are included because the study is ongoing. [b] One of these patients delevop celiac disease
