# Supplementary material for: Proof of Concept of Microbiome-Metabolome Analysis and Delayed Gluten Exposure on Celiac Disease Autoimmunity in Genetically At-Risk Infants
Source: PLoS One. 2012 Mar 14;7(3):e33387. doi: 10.1371/journal.pone.0033387 (PMC3303818; doi:10.1371/journal.pone.0033387)
Supplement: Table S2 — Stool samples collected from each subject. (PDF) [file pone.0033387.s007.pdf]

Table S2. Stool samples collected from each subjects

ID	Group	**A**<br>7d	**B**<br>30d	**C**<br>6m	**D**<br>8m	**E**<br>10m	**F**<br>12m	**G**<br>18m	**H**<br>24m
5	A			5C			5F	5G	
7	A			7C		7E	7F	7G	
9	A			9C	9D	9E	9F		9H
12	A	12A	12B	12C	12D	12E	12F	12G	
13	A	13A	13B	13C	13D	13E	13F	13G	
15	A	15A	15B	15C	15D	15E	15F	15G	
16	A			16C	16D		16F	16G	
22	A	22A	22B	22C	22D	22E	22F	22G	22H
6	B	6A	6B	6C			6F	6G	
3	B	3A	3B	3C			3F	3G	
8	B	8A	8B	8C	8D	8E	8F	8G	
14	B	14A	14B	14C	14D	14E	14F	14G	
17	B	17A	17B	17C	17D	17E		17G	17H
20	B	20A	20B	20C	20D	20E	20F	20G	20H
24	B	24A	24B	24C	24D	24E	24F	24G	
25	B	25A	25B		25D		25F		
