# Supplementary material for: Proof of Concept of Microbiome-Metabolome Analysis and Delayed Gluten Exposure on Celiac Disease Autoimmunity in Genetically At-Risk Infants
Source: PLoS One. 2012 Mar 14;7(3):e33387. doi: 10.1371/journal.pone.0033387 (PMC3303818; doi:10.1371/journal.pone.0033387)
Supplement: Table S3 — Antibody positivity cumulative incidence. (PDF) [file pone.0033387.s008.pdf]

Table S3. Antibody positivity cumulative incidence

Exposure to Gluten	**Group A**	**Group B**
	#/screened (%) Ab positive subjects	#/screened (%) Ab positive subjects
6m	0/13 (0%)	0/13 (0%)
12m	0/12 (0%)[a]	8/13 (61.5%)
18m	1/11 (9.1%)[a]	5/11 (45.5%)[a]
24m	0/10 (0%)[a]	5/9 (55.5%)[a,b]

[a], not all 13 subjects are included because the study is ongoing. [b] One of these patients delevop celiac disease
